# Editorial: Current insights in melanoma immunology, immune escape and immunotherapy advances

**DOI:** 10.3389/fimmu.2026.1868805

**Published:** 2026-05-18

**Authors:** Dimitrios C. Ziogas, Charalampos Theocharopoulos, Pedro Berraondo, Josep M Piulats, Helen Gogas

**Affiliations:** 1 First Department of Internal Medicine, Laikon General Hospital, National and Kapodistrian University of Athens, Athens, Greece; 2 Department of Surgery, University of Colorado Anschutz Medical Campus, Aurora, CO, United States; 3 Program of Immunology and Immunotherapy, Cima Universidad de Navarra, Cancer Center Clínica Universidad de Navarra (CCUN), Pamplona, Spain; 4 Navarra Institute for Health Research (IDISNA), Pamplona, Spain; 5 Centro de Investigación Biomédica en Red de Cáncer (CIBERONC), Madrid, Spain; 6 Medical Oncology Department, Institut Català d’Oncologia. L’Hospitalet del Llobregat, Barcelona, Spain; 7 Cancer Immunotherapy (CIT) Group - iPROCURE, IDIBELL-OncoBell, L’Hospitalet del Llobregat, Barcelona, Spain; 8 Centro de Investigación Biomédica en Red de Cáncer - CIBERONC, Madrid, Spain

**Keywords:** immune resistance mechanisms, immune checkpoint inhibitors (ICIs), immune-related adverse events (irAE), immunotherapy, melanoma, predictive biomarker

Melanoma is a highly aggressive malignancy characterized by a strong immunological background that critically influences disease progression and clinical outcomes. Its intrinsic immunogenicity, combined with insights into T-cell co-inhibitory signaling, provided the mechanistic rationale for immune checkpoint inhibition (ICI). The introduction of ICIs has fundamentally altered the natural history of advanced disease by reinvigorating antitumour T-cell activity through blockade of inhibitory signaling pathways. Despite these advances, a substantial proportion of patients do not respond or develop melanoma relapse, reflecting complex and multifactorial mechanisms of immunotherapy resistance (1). Contemporary research is increasingly focused on defining the cellular and molecular determinants of immune evasion and therapeutic resistance, on recognizing pre-treatment biomarkers predicting immune response and developing novel immunotherapeutic agents that will prolong patient survival, while the overgrowing spectrum of immune-related toxicities remain always a noticeable issue (1, 2).

Programmed cell death (PCD) pathways have emerged as important modulators of antitumor immunity. Induction of immunogenic cell death has been explored as a strategy to activate both innate and adaptive immune responses, although clinical evidence remains limited (1). In their review, Shan and Liu synthesize non-apoptotic PCD pathways, outlining their molecular regulation and immunological consequences in melanoma. Ferroptosis, characterized by iron-dependent lipid peroxidation, has been associated with increased tumour immunogenicity and the release of damage-associated molecular patterns; pyroptosis promotes inflammatory cytokine release, facilitating immune cell recruitment; and necroptosis has been linked to enhanced antigen presentation and activation of adaptive immune responses. The authors propose that modulation of these pathways may enhance responses to ICI, while emphasizing limitations that currently constrain clinical translation. In the same concept, Hu et al. investigate the role of programmed cell death–associated transcriptional signatures in shaping the melanoma tumor microenvironment and clinical outcomes. Using single-cell and bulk transcriptomic analyses, they developed a 15-gene PCD-related prognostic model that stratifies patients into risk groups with distinct survival outcomes. Low-risk tumors were characterized by higher tumor mutational burden, greater immunogenicity and favorable immune infiltration, including CD8^+^ T cells and M1 macrophages, whereas high-risk tumors exhibited immune exclusion, cancer-associated fibroblast enrichment, and immunosuppressive features. The model correlated with immune checkpoint expression and was associated with features linked to immunotherapy responsiveness, and showed consistent prognostic performance across external ICB-treated cohorts, suggesting that PCD-associated transcriptional states reflect tumor–immune interactions relevant to outcome.

The biological heterogeneity of melanoma extends beyond the skin, the disease immune microenvironment varies across anatomical sites, and brain metastasis represents a clinically and immunologically distinct context (3). Zhou et al. performed whole-exome sequencing of tumor and adjacent non-cancerous tissue from three patients with primary malignant melanoma of the cervix, identifying a predominant C>T/G>A substitution pattern, previously unreported mutated genes, and copy-number gains that may activate NFκB and AKT signaling. Claus et al. describe a patient with mucosal melanoma refractory to multiple ICIs who experienced near-complete regression following hepatic embolization and ICI rechallenge, interpreted as a potential abscopal effect. While not generalizable, this observation suggests that locoregional therapy may modulate systemic immune response in selected cases. Najjary et al. used digital spatial profiling to interrogate the immune microenvironment of treatment-naïve primary cutaneous melanomas stratified by subsequent metastatic pattern. In a small cohort of fifteen primary melanomas, tumors from patients who later developed brain metastases demonstrated increased CTLA-4 expression within immune-cell–rich regions and elevated fibronectin expression within tumor-rich regions, compared with tumors that metastasized to extracranial sites. Lower CD137/4-1BB expression was associated more broadly with distant metastatic progression. These findings suggest that spatial immune and extracellular-matrix features in the primary melanoma may be linked to subsequent brain metastatic tropism, although the small sample size warrants cautious interpretation. Complementing these data, Wang et al. provided a comprehensive overview of combined CTLA-4/PD-1 blockade in melanoma brain metastases, discussing meaningful intracranial responses in asymptomatic patients, alongside challenges including optimal sequencing, resistance, toxicity, and blood–brain barrier–related constraints.

Reliable pre-treatment prediction of immunotherapy benefit remains an unmet need in melanoma (4). De Summa et al. used serum ¹H-NMR metabolomics in 71 anti–PD-1-treated metastatic melanoma patients and identified metabolomic signatures associated with progression-free and overall survival; glucose was consistently associated with adverse outcomes, whereas glutamine and free HDL cholesterol contributed to more favorable prognosis, with higher concordance indices observed in the first-line ICI subset. van Dijk et al. evaluated exhaled volatile organic compound profiling using an electronic nose in the prospective melaNose feasibility study, and reported 88% sensitivity, 79% specificity, and 85% accuracy for identifying patients who will not benefit from first-line treatment with ICIs. In a multiparameter flow cytometric analysis, Palli et al. did not found numerical differences in the circulating immune-cells between patients with stage III and stage IV melanoma and between long responders and early progressors. However, DEA and GO results retrospectively identified differences in the transcriptional profiles of pre-treatment peripheral CD4^+^ T-cells between long responders and early progressors, observed in both stage III and stage IV melanoma, showing that melanoma patients could be molecularly clustered separately in advance and that peripheral CD4^+^ T-cell profiling could have a predictive role in ICI response. While systemic biomarkers provide indirect measures of treatment response, tumor immune architecture remains a key determinant of outcome (5). Cai et al. combined bulk and single-cell transcriptomics to define a KRAS–macrophage-associated gene signature linked to survival and immune contexture. High-risk scores were associated with adverse outcomes and features suggestive of reduced immunotherapy responsiveness. Functional silencing of CLEC4A in melanoma cell lines promoted proliferation, migration and invasion. Complementing this, Barrio-Alonso et al. demonstrated that inflammatory cytokine–producing tumor-associated macrophages are enriched in tumors with metastatic progression and are associated with adverse outcomes, while tumor-cell PD-L1 expression emerges as an independent adverse prognostic factor for disease-free and overall survival. In contrast, overall myeloid cell density did not correlate with distant metastasis.

Immune-related adverse events remain a central challenge in the overgrowing clinical use of immune checkpoint inhibitors (6). Strouse et al. show that steroid-sparing immunosuppressive strategies may be associated with worse survival outcomes, particularly with TNF-α inhibitors, although causation cannot be established. Sánchez-Camacho et al. describe myocarditis–myositis–myasthenia gravis overlap syndrome, a rare but severe toxicity with in-hospital mortality rates of 40–60%. Gu et al. report a patient with camrelizumab-associated type I renal tubular acidosis attributed to immune-related connective tissue disease; resolution required potassium citrate and hydroxychloroquine, with corticosteroids being deliberately avoided. Recurrence upon rechallenge led to permanent treatment discontinuation. Together, these studies demonstrate that immune-related toxicity is a major determinant of therapeutic strategy and clinical outcome.

Summarizing, the contributions in this Research Topic highlight the complexity of melanoma immunobiology, spanning mechanisms of immune evasion, tumor–microenvironment interactions, and emerging strategies for response prediction and toxicity management. The editors of this Research Topic would like to thank all authors for their valuable contributions and reviewers for their thoughtful evaluations and support throughout the review process. We hope that this Research Topic provides meaningful insights into melanoma immunology and contributes to ongoing research and clinical advances in the field.
